# Trends in diabetes medication prescribing from 2018 to 2021: A cross-sectional analysis

**DOI:** 10.1371/journal.pone.0307451

**Published:** 2024-08-15

**Authors:** Jessica Riad, Fred Abdelmalek, Noah Ivers, Mina Tadrous

**Affiliations:** 1 Leslie Dan Faulty of Pharmacy, University of Toronto, Toronto, ON, Canada; 2 Women’s College Hospital, Toronto, ON, Canada; Royal College of Surgeons in Ireland, IRELAND

## Abstract

Several new classes of medications for diabetes have recently become available newer medication classes have been increasing in use. It is unclear how their utilization varied across provinces and how the COVID-19 pandemic may have affected these trends. Our objective was to investigate Canada-wide and province-specific trends in diabetes medication dispensed by drug class over time, while also examining the impact of the COVID-19 pandemic and related restrictions on diabetes medication dispensing. We conducted a repeated cross-sectional analysis study. Data were obtained from IQVIA’s CompuScript database for Canada-wide prescription dispensing patterns in primary care from January 2018 to December 2021. Drug classes of interest were biguanides dipeptidyl peptidase 4 inhibitors, sulfonylurea’s, insulins, sodium-glucose co-transporter 2 inhibitors, and glucagon-like peptide-1 receptor agonists. We examined trends before and after the onset of the pandemic with special attention to changes during periods of high COVID-19 activity. Most drug classes displayed a stable number of prescriptions each month throughout, except for glucagon-like peptide-1 receptor agonists and sodium-glucose co-transporter 2 inhibitors, which demonstrated a consistent pattern of increased dispensing. Sodium-glucose co-transporter inhibitors and glucagon-like peptide-1 receptor agonists exhibited the greatest growth over the examined period, of 7.9% and 5.0% increases, respectively. For sodium-glucose co-transporter 2 inhibitors, Prince Edward Island (4.0%) displayed the greatest growth while Ontario showed the least (2.5%). For glucagon-like peptide-1 receptor analogs, Saskatchewan (11.3%) displayed the greatest growth and Newfoundland the least (4.5%). The pandemic did not impact overall dispensing trends. However, spikes in COVID-19 cases corresponded to changes in dispensing for most drug classes. Important variations across Canada in guideline-recommended medication classes seems to be increasing over time. This is likely due to differing formulary listing and access to drug coverages. If so, future research could explore national formulary harmonization across Canada and health outcomes for patients with diabetes.

## Introduction

Diabetes poses a growing burden both in Canada and globally, with approximately 30% of the population in 2022 affected by type 1 diabetes, diagnosed type 2 diabetes, undiagnosed type 2 diabetes, or prediabetes. In 2032, this prevalence is estimated to grow to 33% making it an increasing burden on the Canadian health care system [1]. Uncontrolled and inadequately managed diabetes has micro and macrovascular long-term complications therefore, pharmacotherapy is often required for glucose control [2].

Over the past two decades, the landscape of medication for treatment in Canada has changed with new medications being available to citizens. In year 2020, the Diabetes Canada guidelines recommended use of new classes, namely sodium-glucose co-transporter 2 (SGLT-2) inhibitors and glucagon-like peptide-1 receptor (GLP-1) agonist, as preferred second-line pharmaceutical agents for type 2 diabetes [3]. However, as new medication classes become available to Canadians, they are added to provincial formularies at differing times. In addition to provincial policy causing varying access, the COVID-19 pandemic posed restrictions to availability.

Given the breadth of the pharmacological choices available to clinicians for the management of diabetes, and considering numerous new medications now available to Canadians, we wanted to explore the trends of diabetes medication dispensing patterns nationally and provincially in addition to exploring the impact of the COVID-19 pandemic on these trends. Understanding how dispensing has changed over time and across provinces is crucial for healthcare professionals, policymakers, and researchers to ensure evidence-based and personalized diabetes management across Canada.

## Methods

We conducted a repeated cross-sectional analysis study, using estimated community-based prescription medication dispensing data from IQVIA’s CompuScript dataset between January 2018 to December 2021. The anonymized data was access on June 8^th^, 2022. Importantly, this study is conducted prior to the rapid rise of GLP-1 use for weight loss to allow for an evaluation of the use of these medication before the rapid increase in their off-label use. This study focuses on prescription medications dispensed in outpatient / retail pharmacies, regardless of payer (patient, private insurance, or government insurance). IQVIA’s CompuScript database uses a representative sample of more than 6500 retail pharmacies across Canada (approximately 60% of all retail pharmacies) and represents 75% of the total prescriptions dispensed at the national level [4]. This database is unique as it includes an estimated record of prescription transactions from Canadian drug stores for both branded and generic medications on a monthly basis. The reliability of the IQVIA database has been previously validated [5, 6]. The COVID- 19 data corresponding to the peaks was retrieved from published newspaper articles and analyzed to create a COVID-19 infection timeline table [7]. We report diabetes medication dispensing results for the six following drug classes available in Canada: biguanides, dipeptidyl peptidase 4 (DPP-4) inhibitors (alone or combination), sulfonylureas (SU’s), insulins, SGLT-2 inhibitor, and GLP-1 agonists. These drug classes were chosen and analyzed in the cross-sectional analysis based on them being the approved and the major drug classes utilized by the Canadian population for treatment of diabetes. The data was stratified by province and month to determine heterogeneity in dispensing across geographical location and time.

## Results

**Fig 1:**
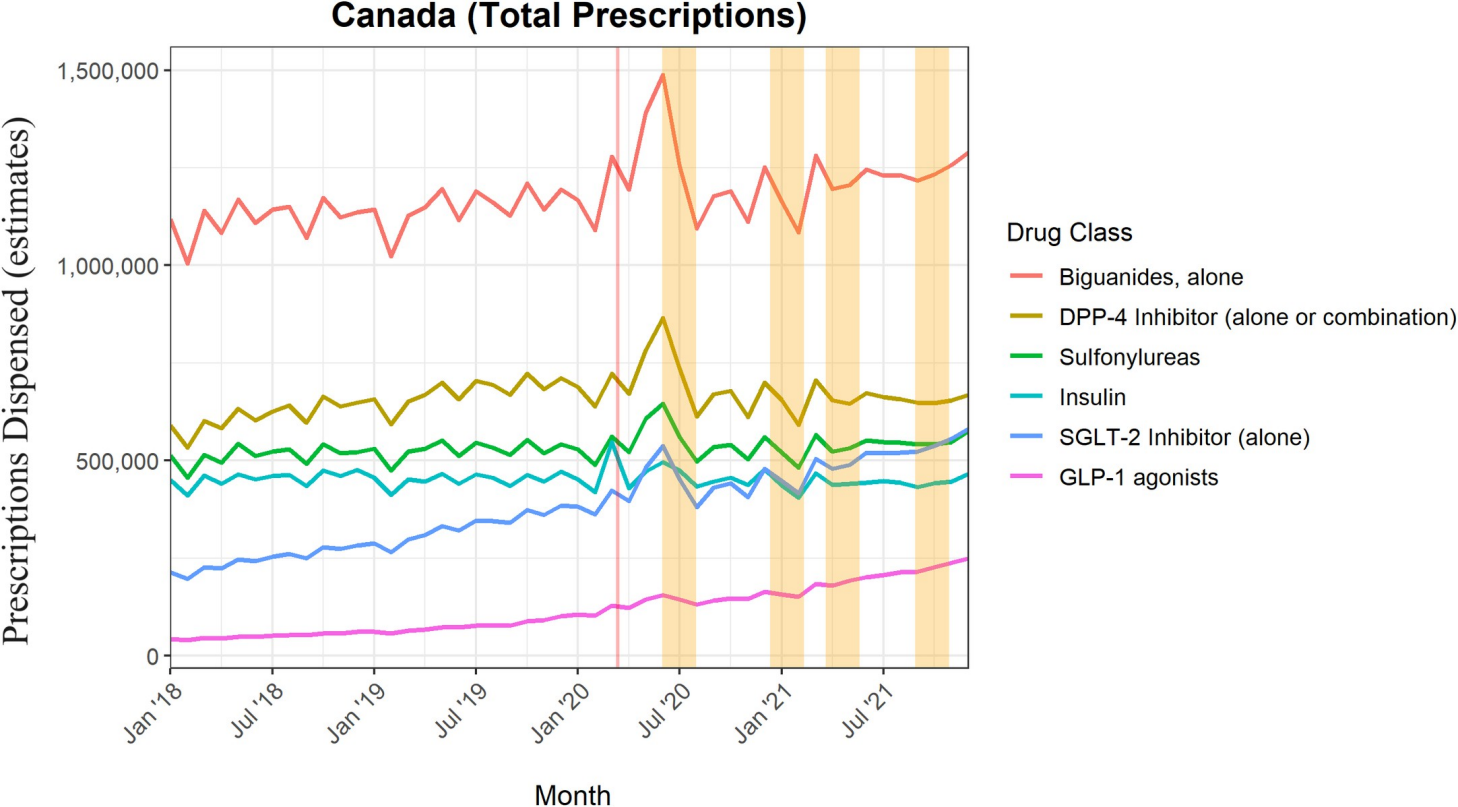
Monthly total prescriptions by drug class. Highlighted areas represent COVID-19 peaks throughout Canada. Note: This is based on information licensed from IQVIA: CompuScript for the period of January 2018 to December 2021 reflecting estimates of real-world activity. All rights reserved.

Estimated total prescriptions, for all included classes of medication, across Canadian retail pharmacies were analyzed. Fig 1 demonstrates the trends of each drug class from January 2018 to December 2021. Most classes showed a generally stable number of prescriptions over time while SGLT-2 inhibitors and GLP-1 analogues demonstrate a steady increase in the number of prescriptions. On average, the most prescribed nationwide medication class was biguanides, followed by DPP-4 inhibitors, SU’s, insulins, SGLT-2 inhibitors, and GLP-1 agonists. Fig 1 displays highlighted areas that represent COVID-19 peaks, as defined by increase in cases nationally, in which most drug classes had a dramatic increase in prescriptions. The red vertical line corresponds to March 11, 2020, which is the day COVID-19 was declared a pandemic by the World Health Organization. The other 4 yellow areas correspond to exactly 60-day intervals centered around the peaks of the first 4 waves of the pandemic. The specific dates of the peaks (i.e., the centers of the 4 yellow regions) are May 30, 2020, January 10, 2021, April 18, 2021, and September, 26 2021 [7].

In January 2018, biguanides made up 38.2% of all prescriptions, DPP-4 Inhibitors (alone or in combination) 20.2%, SU’s 17.5%, insulin 15.4%, SGLT-2 inhibitors 7.3%, and GLP-1 analogs 1.5%. Conversely, in December 2021, biguanides made up 33.7% (-4.5%), DPP-4 inhibitors 17.4% (-2.8%), SU’s 15% (-2.5%), insulin 12.2% (-3.2%), SGLT-2 inhibitors 15.2% (+7.9%), and GLP-1 analogs 6.5% (+5%). SGLT-2 inhibitors exhibited the greatest growth over the assessed time frame and surpassed SU’s and insulins by December 2021.

The growth of prescribed SGLT-2 inhibitors and GLP-1 analogs from January 2018 to December 2021 is also reflected in Table 1. Table 1 reports the factor by which prescriptions for these medications grew in each province. The growth of each province varied between 2.47 to 4.01 for SGLT-2 inhibitors and 4.47 to 11.31 for GLP-1 analogs growth. For SGLT-2 inhibitors, Prince Edward Island (4.01) displayed the greatest growth and Ontario showed the least (2.47). For GLP-1 analogs, Saskatchewan (11.31) displayed the greatest growth and Newfoundland the least (4.47).

**Table 1:** Growth of SGLT-2 inhibitors and GLP-1 agonists by province. Note: This is based on information licensed from IQVIA: CompuScript for the period of January 2018 to December 2021 reflecting estimates of real-world activity. All rights reserved.

Province	SGLT-2 Growth (Dec 2021 / Jan 2018)	GLP-1 Growth (Dec 2021 / Jan 2018)
Alberta	2.52	5.50
British Columbia	3.04	4.87
Manitoba	3.34	8.84
New Brunswick	2.68	6.39
Newfoundland	3.42	4.47
Nova Scotia	3.95	4.33
Ontario	2.47	7.64
Prince Edward Island	4.01	5.80
Quebec	2.93	4.82
Saskatchewan	2.89	11.31
Canada	2.72	5.86

**Fig 2:**
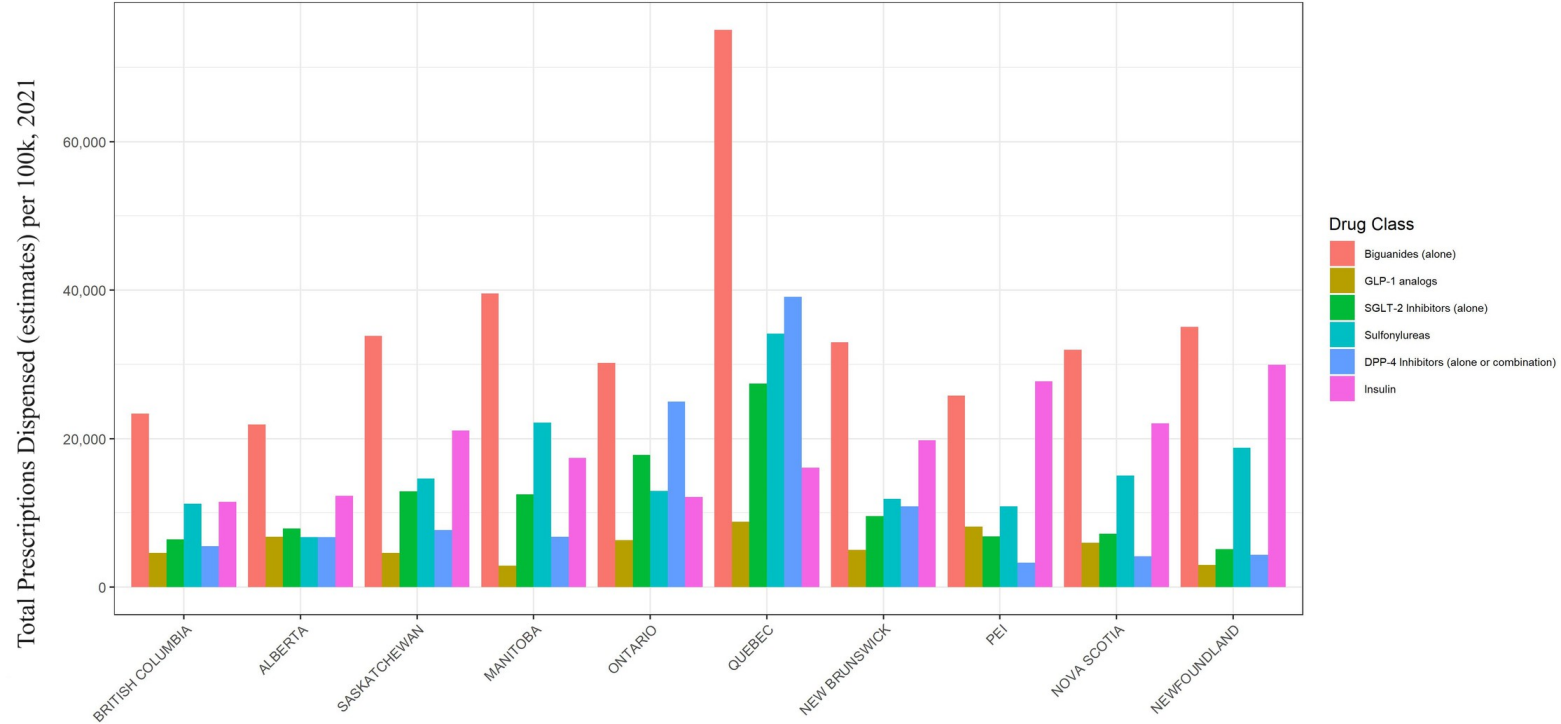
Prescriptions by province and drug class (for 2021). Note: This is based on information licensed from IQVIA: CompuScript for the period of January 2018 to December 2021 reflecting estimates of real-world activity. All rights reserved.

In Fig 2, the number of prescriptions for each drug class in 2021 is plotted by province. The most prescribed medication across all provinces is biguanides alone, with differing prescription totals among provinces. Quebec surpasses all other provinces for biguanide prescriptions total. This is also true for all the other drug classes apart from insulin.

## Discussion

We explored the trends in diabetes medication dispensing across Canada by drug class between January 2018 and December 2021. For most drug classes, the number of prescriptions dispensed each month remained roughly similar over time with biguanides remaining the most common drug class throughout this timeframe. For GLP-1 analogs and SGLT-2 inhibitors, the number of monthly prescriptions across the nation rose significantly, both in absolute terms and relative to the other drug classes. The rise in GLP-1 analogs and SGLT-2 inhibitors prescriptions parallels the age-standardized incidence of diabetes that has increased yearly from 2018 to 2020 [8]. Incidence during the pandemic was less than in 2018–2019 and 2019–2020, at 497 per 100,000, which may a result of Canadians altering their health seeking behaviours and a change in health service availability.

During COVID-19 waves, there were increases in dispensing for most drug classes demonstrating that access to medication was not greatly altered by the pandemic. Surges in dispensing associated with the COVID-19 waves may be a result of policy altercations to adapt to supply and demand concerns [8]. For example, in Ontario, prescribers were limited to a 30- day supply of prescriptions for all Ontario Drug Benefit users. A similar regulation applied to most of the provinces and territories (Alberta, Saskatchewan, Manitoba, Ontario, Nova Scotia, Prince Edward Island, Newfoundland, Yukon) for varying time frames throughout the pandemic [9]. Despite the dramatic spikes in dispensing, the national trends for dispensing were not altered.

There was interprovincial variation in dispensing for all drug classes, with some provinces experiencing noteworthy increases in GLP-1 analogs and SGLT-2 inhibitors. Interestingly, Manitoba exhibited the highest diabetes incidence during the 2020–2021 fiscal year (721 per 100,000), yet this province did not experience the most significant expansion in overall prescriptions or the growth of GLP-1 analogs and SGLT-2 inhibitors [8]. Interprovincial variation has been found in other medications, such as antibiotics and psychotropics indicating an inequity among other drugs [10, 11].

Access to insurance also varies among provinces with 26% of British Columbia residents without any prescription coverage in 2021. Even greater was 33% of British Columbia residents who are seniors reporting no coverage during the pandemic. They have the greatest proportion of residents without insurance to cover medication costs while Nova Scotia has the least (14%) [12]. For Canadians with drug insurance plans, it varies between government sponsored plans, employer- sponsored plans, association sponsored plans, and private plans. Provinces range in distribution however, in 2019, Quebec had the greatest proportion of men and women with any type of coverage (88.8%) [13]. Specifically for private drug coverage, Ontario had the least amount of private sponsored plans of 4.1% in 2019 while Saskatchewan had the greatest of 15.6% for men and 13.0% for women [13]. Private and public coverage plays a role in medication access as those with private insurance often get access to newly approved medication earlier than public coverage patients. This inequity may play a role in interprovincial variation and does not align with “portability” as per the Canadian Health Act of Diabetes [14].

The emergence of GLP-1 analogs and SGLT-2 inhibitors may be attributed to a shifting landscape of treatment for diabetes and obesity across Canada. As the field progresses with further research on these medications benefits beyond glycemic control, these drugs are progressively being prescribed to a greater number of people nationally [15]. With increased knowledge on these medications’ cardiovascular benefits supplementary to the strong evidence on its other health benefits and listing to public formularies, there was an emergence of GLP-1 analogs with a rapid rise in 2021. Canagliflozin was the first SGLT-2 inhibitor added to the Canadian formulary in 2014 however, to date, there still remains SGLT-2 inhibitor medication restricted in some province formularies [16, 17]. Similar patterns follow for GLP-1anlogs with varying restrictions since the first GLP-1 agonist, liraglutide, was added to formulary in 2010 [17, 18] More recently, semaglutide pills were approved in 2020 but only Ontario has it listed in their formulary as a diabetes medication, while the other provinces and territories do not [17, 18]. The data we used is derived from a time period prior to the proliferation of GLP-1 analogs prescribing and usage for both obesity and diabetes. Therefore, our analysis for the years 2018 to 2021, focus largely on its utilization specifically for diabetes patients.

In contrast to existing Canadian literature examining trends in diabetes medication, this article introduces novel insights. Carney et al.’s 2022 study, which specifically investigated oral glucose-lowering agents in British Columbia, similarly identified biguanides as the most frequently prescribed agents among their cohort [19]. Our article broadens the scope to include insulin as a glucose-lowering agent and examines trends across multiple provinces rather than focusing solely on one. Secrest and colleagues explored comparable trends across four Canadian provinces; however, their dataset only extended up to 2014, missing the emergence of GLP-1 analogs in prescribing practices for both diabetes and obesity [20]. This study has many strengths. Firstly, its dataset is unique as it encompasses both public and private payers for medications across Canada and by province. This inclusivity provides an accurate depiction of the evolving landscape of pharmacotherapy for diabetes. It covers 75% of prescriptions nationwide, enhancing the generalizability of the results to the entire nation. Secondly, the study spans a substantial timeframe, capturing data from both pre- and post-pandemic periods. Additionally, the data is stratified among provinces, allowing for a more in-depth analysis of specific trends. Lastly, this study stands out for its uniqueness and significance in addressing the impact of the pandemic on specific Canadian provinces and national trends, a topic that has yet to be discussed in the literature.

This study has a couple of limitations. Firstly, the data lacks details on clinical or sociodemographic aspects, focusing solely on the quantity of prescriptions dispensed rather than directly monitoring patients. This omission hinders our ability to assess how patient characteristics might restrict medication access and influence prescribing patterns. Secondly, the dataset does not consider polypharmacy across all drug classes, limiting our comprehension of the clinical implications associated with these trends. Furthermore, the data represents dispensed prescriptions, with no indication of the total number of individuals involved. Consequently, although we assume most prescriptions are for individuals with type 2 diabetes, the specific diagnoses remain unknown.

## Conclusions

Overall, the number of prescriptions for each medication class remained roughly similar or slowly grew. However, GLP-1 analogs and SGLT-2 inhibitors grew rapidly in the study period. Waves of the COVID-19 pandemic were associated with increased prescription dispensing but did not impact overall national trends. Heterogeneity in the management of diabetes within the Canadian healthcare system may reflect inconsistent formulary determinations. Consequently, our results provides an opportunity to explore the need for comprehensive harmonization across Canada and explore how differing access to care may be impacting diabetes outcomes.

## Supporting information

S1 Fig Proportion of prescriptions by province (bars). Note: This is based on information licensed from IQVIA: CompuScript for the period of January 2018 to December 2021 reflecting estimates of real-world activity. All rights reserved.
(TIF)

S2 Fig Number of prescriptions for each drug class by province. Note: This is based on information licensed from IQVIA: CompuScript for the period of January 2018 to December 2021 reflecting estimates of real-world activity. All rights reserved.
(TIF)

S3 Fig Number of prescriptions for each drug class by province, relative. Note: This is based on information licensed from IQVIA: CompuScript for the period of January 2018 to December 2021 reflecting estimates of real-world activity. All rights reserved.
(TIF)

S4 Fig Proportion of monthly total prescriptions contributed by each drug class. Note: This is based on information licensed from IQVIA: CompuScript for the period of January 2018 to December 2021 reflecting estimates of real-world activity. All rights reserved.
(TIF)
