# Supplementary figures and images for: Trends in diabetes medication prescribing from 2018 to 2021: A cross-sectional analysis
Source: PLoS One. 2024 Aug 15;19(8):e0307451. doi: 10.1371/journal.pone.0307451 (PMC11326546; doi:10.1371/journal.pone.0307451)

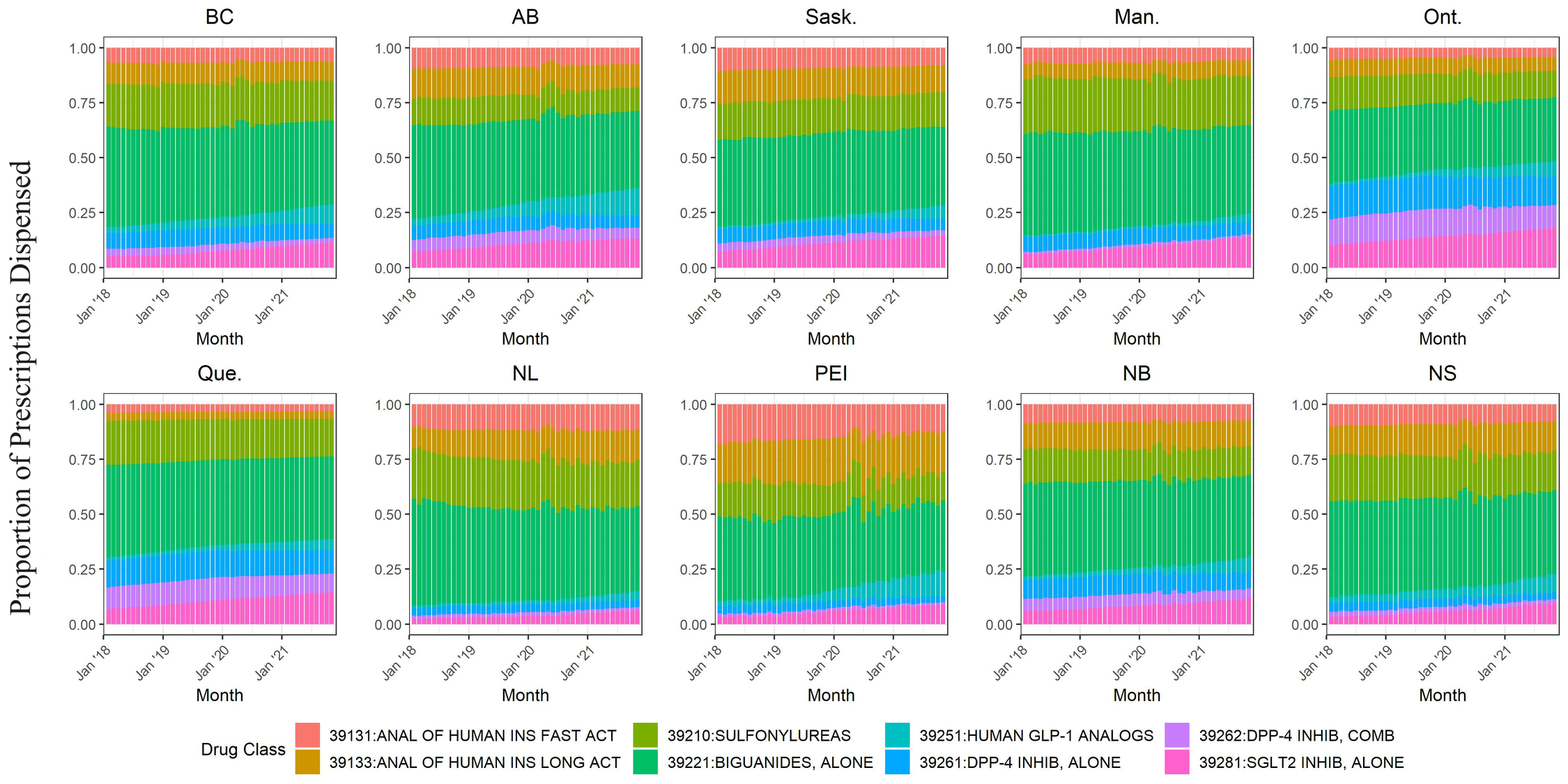

Supplement: S1 Fig — Note: This is based on information licensed from IQVIA: CompuScript for the period of January 2018 to December 2021 reflecting estimates of real-world activity. All rights reserved. (TIF) [file pone.0307451.s001.tif]

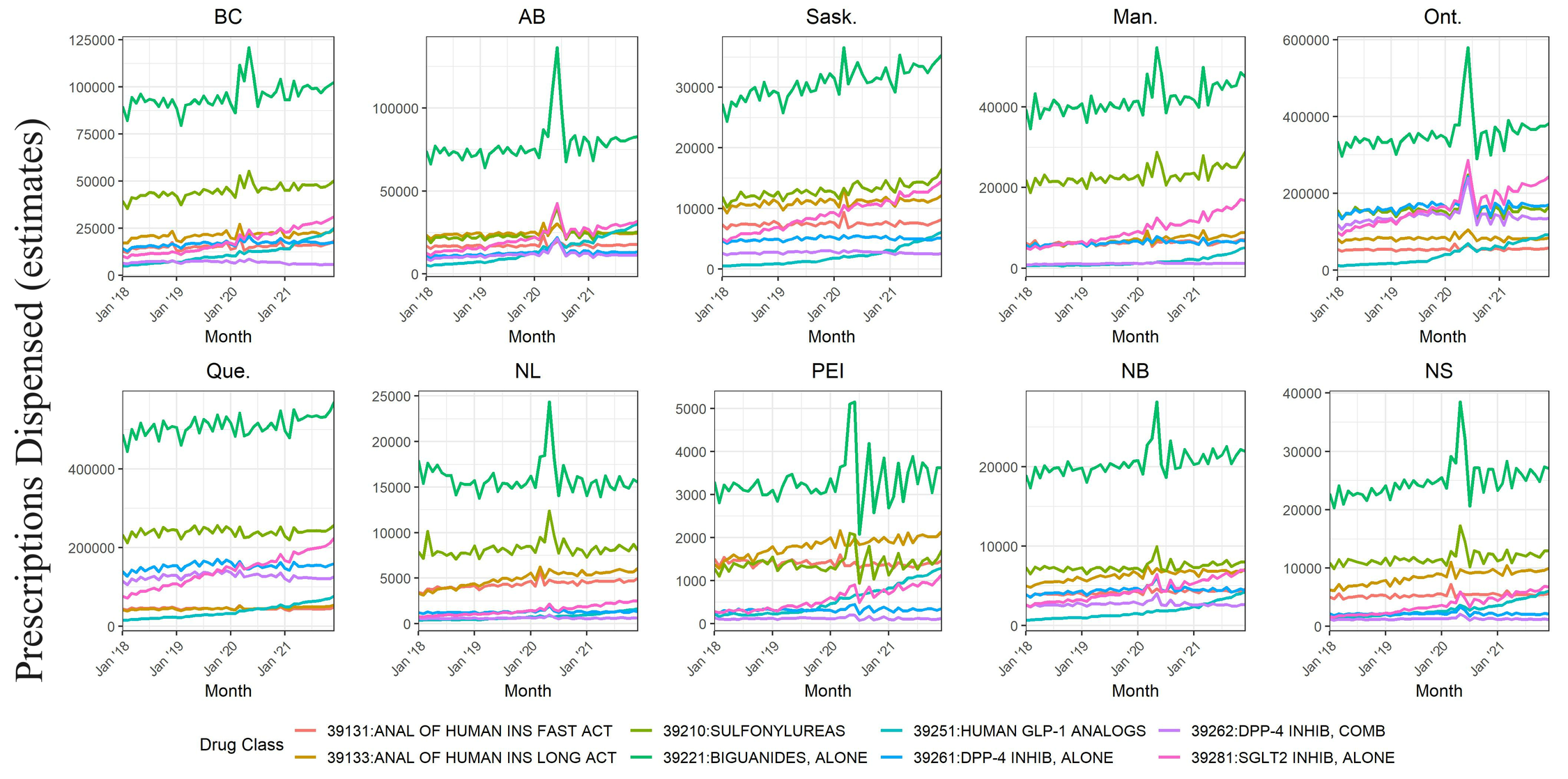

Supplement: S2 Fig — Note: This is based on information licensed from IQVIA: CompuScript for the period of January 2018 to December 2021 reflecting estimates of real-world activity. All rights reserved. (TIF) [file pone.0307451.s002.tif]

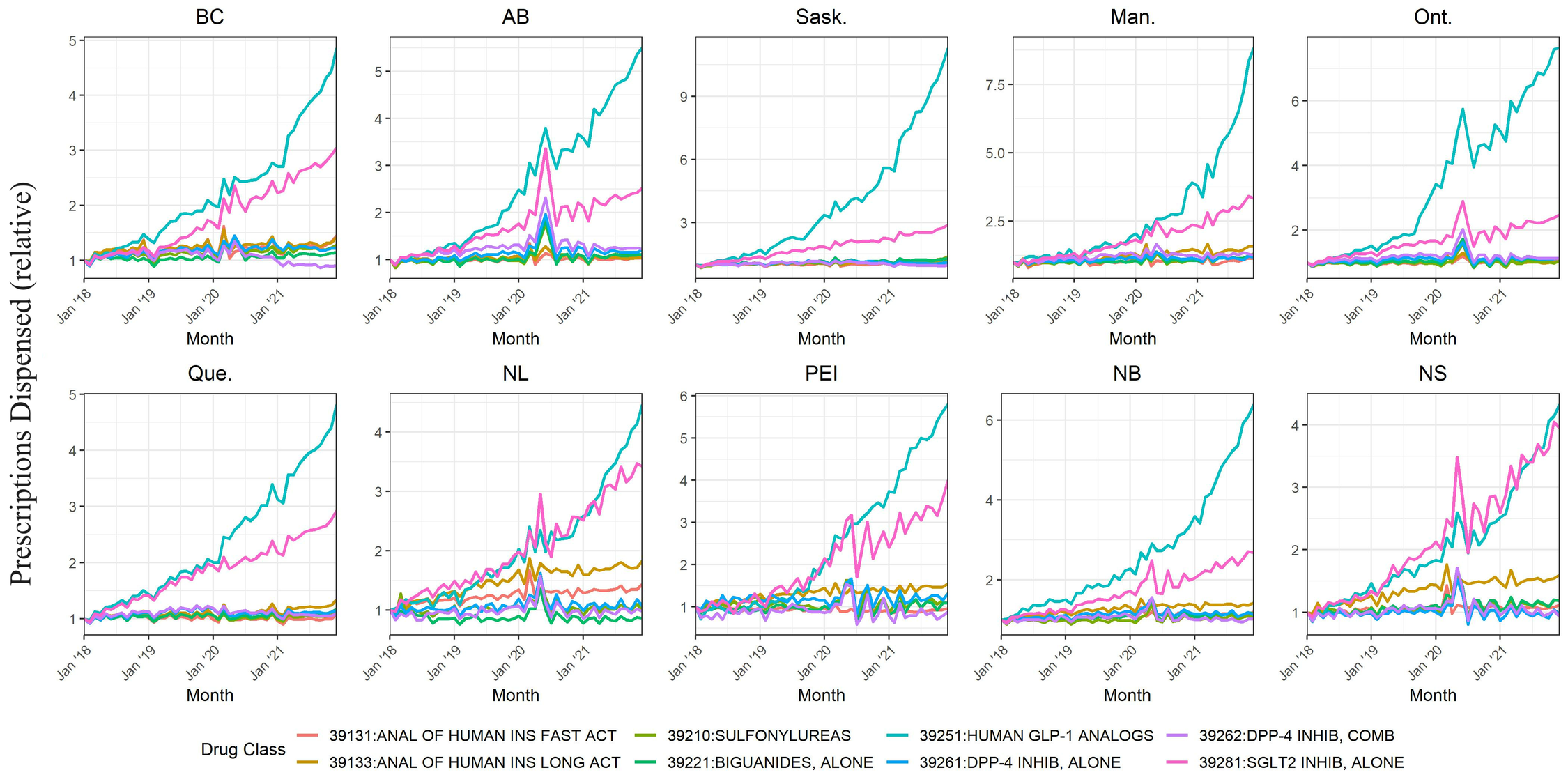

Supplement: S3 Fig — Note: This is based on information licensed from IQVIA: CompuScript for the period of January 2018 to December 2021 reflecting estimates of real-world activity. All rights reserved. (TIF) [file pone.0307451.s003.tif]

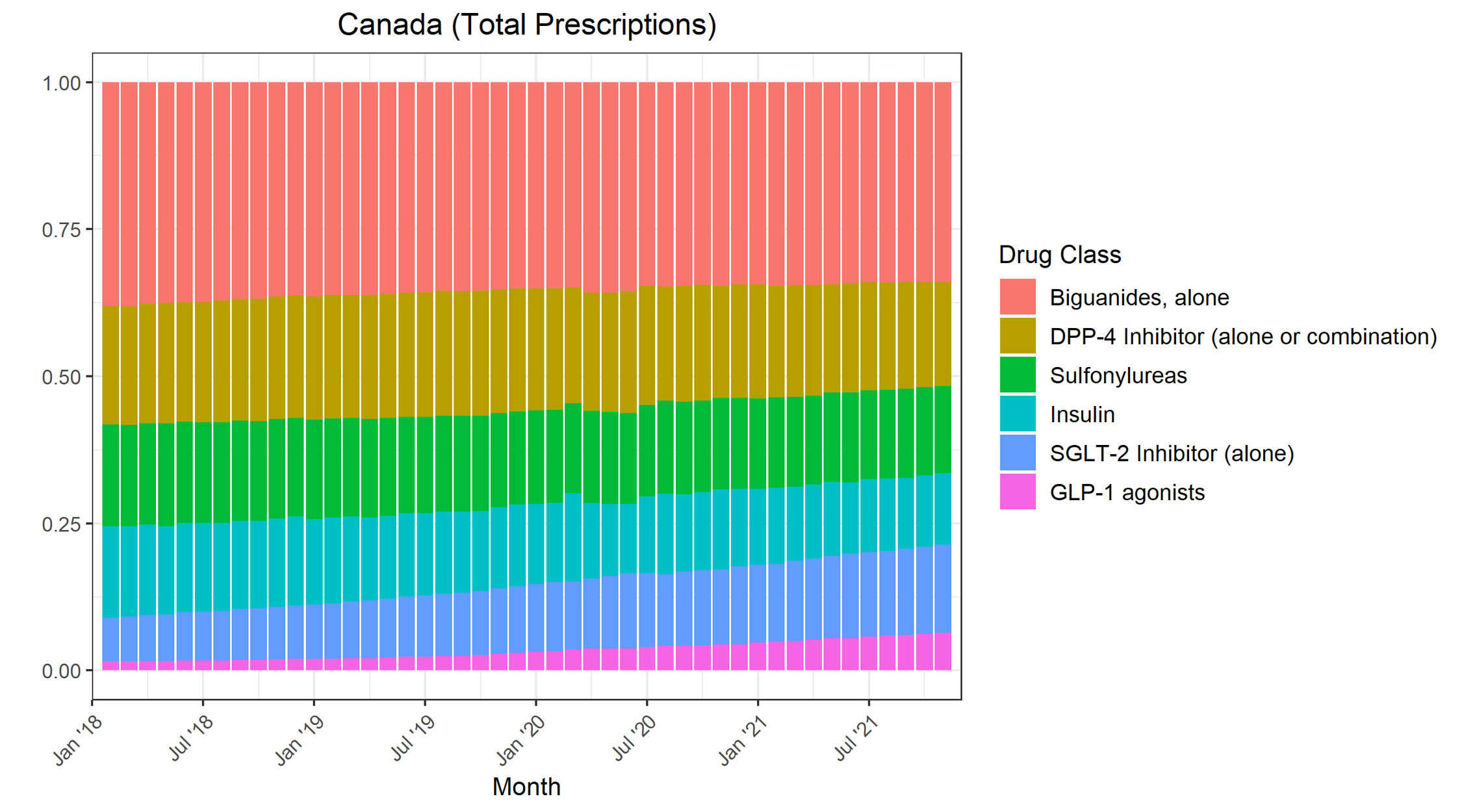

Supplement: S4 Fig — Note: This is based on information licensed from IQVIA: CompuScript for the period of January 2018 to December 2021 reflecting estimates of real-world activity. All rights reserved. (TIF) [file pone.0307451.s004.tif]
